# A case of pulmonary mucosa-associated lymphoid tissue (MALT) lymphoma in a patient with a history of idiopathic lymphocytic interstitial pneumonia (iLIP)

**DOI:** 10.1186/s44215-025-00208-3

**Published:** 2025-05-09

**Authors:** Katsushi Toyohara, Hiroya Ishihara, Takuro Morita, Yuki Shindo, Sho Takeda, Satoshi Fumimoto, Kaoru Ochi, Yoshio Ichihashi, Kiyoshi Sato, Hiroko Kuwabara, Nobuharu Hanaoka, Takahiro Katsumata

**Affiliations:** 1 Department of Thoracic Surgery, Hirakata Municipal Hospital, Osaka, Japan; 2 Department of Thoracic Surgery, Kishiwada City Hospital, Osaka, Japan; 3 Department of Thoracic Surgery, Yao Tokushukai General Hospital, Osaka, Japan; 4 Department of Thoracic Surgery, Takatsuki Red Cross Hospital, Osaka, Japan; 5 Department of Thoracic and Cardiovascular Surgery, Osaka Medical and Pharmaceutical University, Osaka, Japan; 6 Department of Thoracic Surgery, Hokusetsu General Hospital, Osaka, Japan; 7 Department of Pathology, Osaka Medical and Pharmaceutical University, Osaka, Japan

**Keywords:** Primary pulmonary lymphoma (PPL), Pulmonary mucosa-associated lymphoid tissue (MALT) lymphoma, Extranodal marginal zone lymphoma (EMZL), Idiopathic interstitial pneumonias (IIPs), Idiopathic lymphocytic interstitial pneumonia (iLIP)

## Abstract

**Background:**

Pulmonary mucosa-associated lymphoid tissue (MALT) lymphoma and idiopathic lymphocytic interstitial pneumonia (iLIP) are rare pulmonary diseases. MALT lymphoma is an extranodal marginal zone lymphoma (EMZL), whereas LIP is a benign lymphoproliferative disorder characterized by lymphocytic infiltration of the lungs. LIP should be closely monitored, as it has the potential to undergo malignant transformation into MALT lymphoma.

**Case presentation:**

A 45-year-old woman was diagnosed with LIP and followed up for 9 years before being referred to our hospital due to an enlarging shadow on chest radiographs. The volume of the sample collected via bronchoscopy was too small to make a diagnosis, so the patient underwent surgery. The pathology results revealed diffuse proliferation of medium-sized lymphocytes filling the alveolar spaces, leading to a diagnosis of MALT lymphoma. After a thorough examination, no other lesions were found, confirming the diagnosis of EMZL of the lung, a primary pulmonary lymphoma (PPL). No postoperative treatment was administered after surgery; however, 2 years later, recurrence was detected in the stomach, and the patient underwent chemotherapy. Complete remission was achieved through chemotherapy, and the patient has been recurrence-free for 3 years since her treatment.

**Conclusions:**

We report a rare case of MALT lymphoma that developed 9 years after the diagnosis of LIP. Since LIP can undergo malignant transformation into EMZL, it is important to be aware of this possibility. Differentiating between the two diseases onthe basis ofclinical and imaging findings is challenging, so biopsytechniques, such as transbronchial biopsy, CT-guided needle aspiration biopsy, and surgical resection, are essential. While surgery is the standard treatment for primary pulmonary lymphoma, observation is a viable option, as it provides results comparable to those of other treatment approaches.

**Supplementary Information:**

The online version contains supplementary material available at 10.1186/s44215-025-00208-3.

## Background

Mucosa-associated lymphoid tissue (MALT) lymphoma was first described in 1983 and is now recognized as a distinct type of non-Hodgkin lymphoma [1]. The primary sites of MALT lymphoma include the stomach, the ocular adnexa, salivary glands, skin, lungs, breasts, thyroid, and thymus [2]. Primary pulmonary lymphoma (PPL) is a rare clinical entity, that accounts for less than 1% of all lymphomas, and pulmonary extranodal marginal zone lymphoma (EMZL) is the most common form of PPL, even though it accounts for less than 1% of all pulmonary malignancies [3]. In contrast, lymphocytic interstitial pneumonia (LIP) was first described in 1966 as a benign lymphoproliferative disorder that is confined to the lungs and characterized by diffuse infiltration of the alveolar septa with dense collections of lymphocytes, plasma cells, and other cellular elements [4]. Several reports in the literature indicate that LIP can undergo malignant transformation into MALT lymphoma. A case of pulmonary MALT lymphoma, which was initially diagnosed as idiopathic LIP (iLIP), is described here.

## Case presentation

**Fig. 1:**
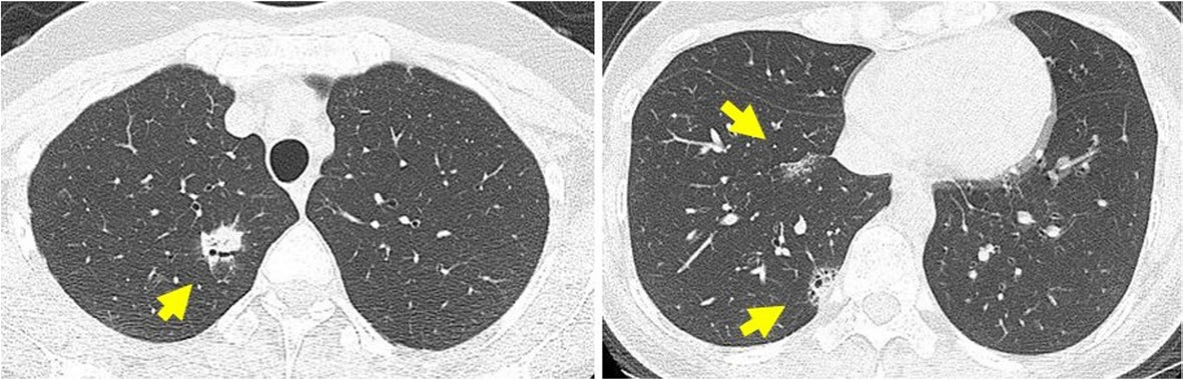
**a** An infiltrative shadow with an air bronchogram in the right S2 segment. **b** CT image showing ground-grass opacity and cystic components in the shadows of the right S8 and S10 segment

**Fig. 2:**
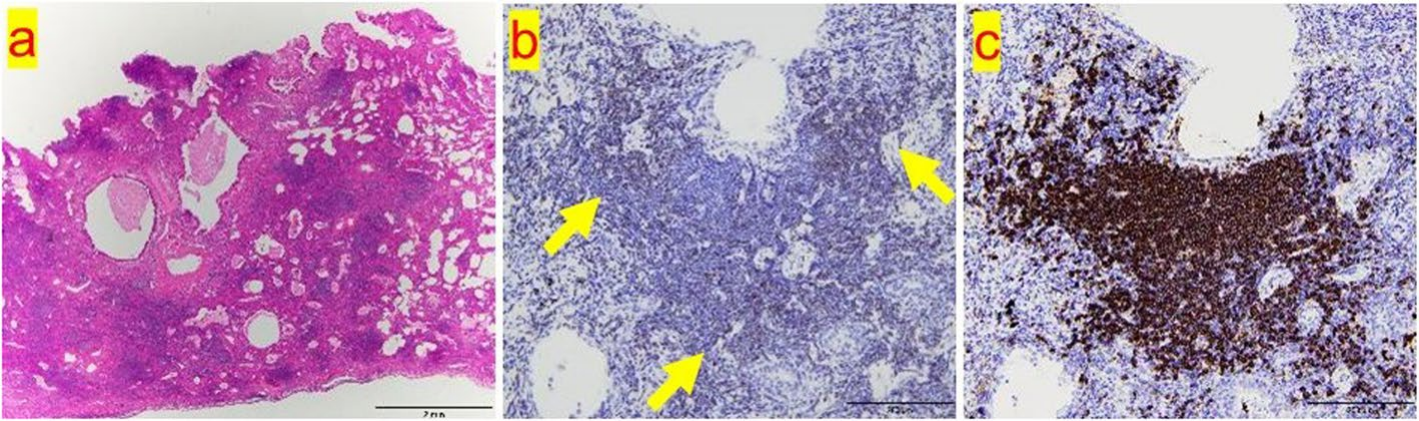
**a** A diffuse lymphoid infiltrate with reactive follicles. The infiltrating lymphocytes are small with little atypia, and lymphoepithelial lesions are absent. **b**, **c** A mixture of small lymphocytes positive for CD3 (b) and CD20 (C) was present

**Fig. 3:**
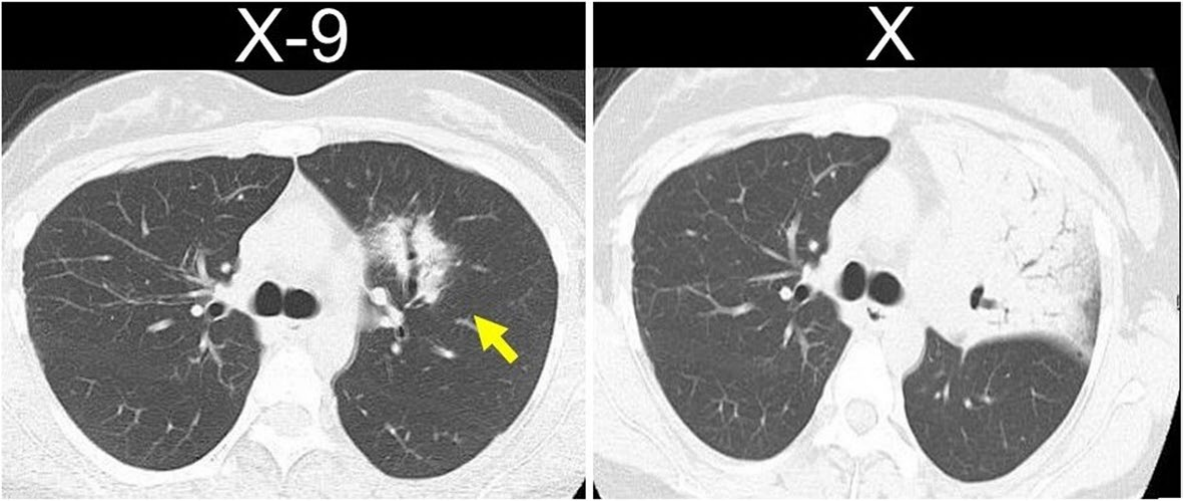
**a** CT revealed consolidation with an air bronchogram. **b** An enlarged shadow was observed. The air bronchogram remained clear, and no evidence of bronchial obstruction was observed

**Fig. 4:**
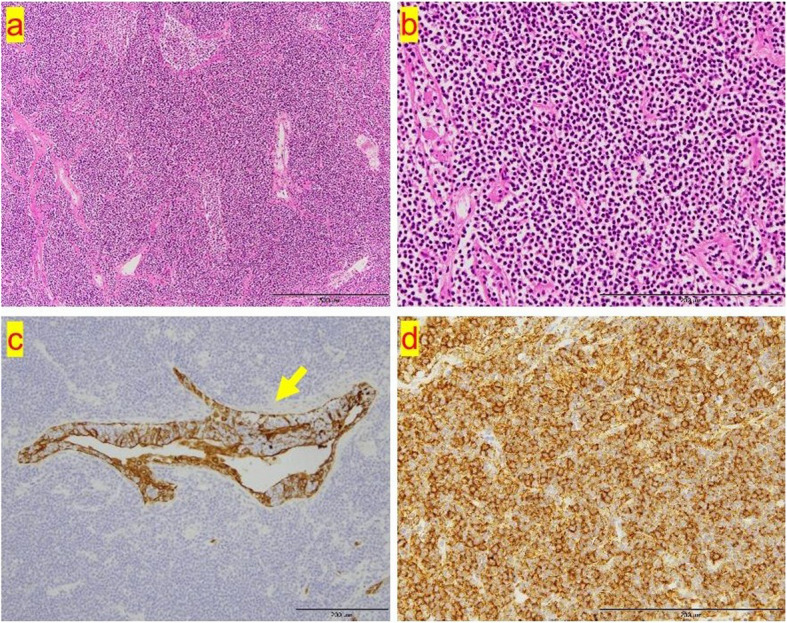
**a**, **b** The pathology results revealed diffuse proliferation of medium-sized lymphocytes filling the alveolar spaces. **c** A lymphoepithelial lesion (LEL) was observed (pan-cytokeratin staining; × 400 magnification). **d** CD20-positive medium-sized lymphocytes were observed

A 36-year-old asymptomatic woman presented to our hospital with abnormal shadows on her chest radiograph during a routine medical check-up. Chest CT revealed multiple shadows in both lungs (Fig. 1a, b), and a bronchoscopy was planned. The lesion in the left upper lobe was the largest, so a biopsy was performed on this lesion. The bronchoscopy findings revealed lymphocytic infiltration of the bronchial mucosa and macrophage exudation in the alveolar spaces (Supplementary material 1). Lymphoma could not be completely excluded; therefore, a surgical biopsy was planned for further evaluation. The patient subsequently underwent video-assisted thoracoscopic surgery (VATS). Given that many of the shadows were thought to be part of the same disease and considering the ease of resection, the shadow in the right lower lobe (S10) was resected. Postoperative pathology findings revealed dense infiltration of small lymphocytes with widened interlobular/alveolar septa and cystic changes (Fig. 2a–c). No autoimmune diseases were identified, and there were no clinical symptoms indicating extrathoracic involvement, or any specific biological findings. Therefore, a diagnosis of iLIP was made. After the patient returned to the referring hospital, steroid treatment was administered for iLIP, but the patient discontinued that treatment and was subsequently monitored. After 9 years of follow-up, the lesion in the left upper lobe gradually enlarged, and the patient was referred back to our hospital (Fig. 3). Chest CT revealed multiple infiltrative shadows in both lung fields. Compared with the previous examination, most of the shadows remained unchanged, with only the shadow in the left upper lobe showing enlargement and extensive atelectasis. The volume of the sample collected by bronchoscopy was too small to make a diagnosis, and surgery was planned. The left upper lobe was nonfunctional due to extensive atelectasis, so a left upper lobectomy was performed. No significant complications were observed during the postoperative course, and the patient was discharged on the 7 th day post-surgery. Postoperative pathology examination revealed diffuse proliferation of medium-sized lymphoid cells resembling centrocyte-like cells (Fig. 4a, b), and these cells formed lymphoepithelial lesions (Fig. 4c). Immunohistochemically, these cells were positive for CD20, CD79a, and BCL-2 but negative for CD3, CD5, CD10, and cyclin D1 (Fig. 4d). Compared with the cells observes in the previous sample, which was biopsied and revealed a diagnosis of iLIP, the CD20-positive lymphocytes in the follow-up sample were medium-sized rather than small, and the lymphoepithelial lesions were prominent. Thus, the patient was diagnosed with EMZL, which was classified as Stage IIE according to the Ann Arbor classification. No other abnormalities were found on the PET scan, so the patient did not receive chemotherapy. However, 2 years after surgery, a new lesion was detected in the stomach, and chemotherapy was administered, resulting in complete remission. The patient was still alive 5 years after surgery.

## Discussion

In 1983, Isaacson and Wright proposed that lymphoma arising from MALT should be classified as MALT lymphoma [1]. The 5 th edition of the WHO classification classifies MALT lymphoma as an EMZL, a type of mature B-cell tumor [2]. Although the incidence of gastric EMZL has decreased with the eradication of *Helicobacter pylori* (H. P) infection, the overall incidence of EMZL has been increasing annually [2]. PPL is defined as clonal lymphoid proliferation involving the lung parenchyma and/or bronchi without detectable extrapulmonary lymphoma at primary diagnosis or for the subsequent 3 months; this disease is extremely rare, accounting for only 0.4% of all lymphomas and less than 0.5% of all primary lung tumors [5]. In contrast, LIP was first reported by Carrington and Liebow in 1966 as a form of interstitial pneumonia characterized by diffuse and marked infiltration of lymphoid cells into the lung interstitium [4]. LIP is assumed to be a secondary condition resulting from various pathological causes, such as collagen diseases, autoimmune diseases, immune abnormalities, and infections [6]. In the absence of the abovementioned underlying conditions, it is classified as idiopathic LIP (iLIP). iLIP was classified as one of the seven major types of idiopathic interstitial pneumonias (IIPs) in the 2002 international interdisciplinary consensus by the ATS/ERS [7]. However, owing to its rarity, iLIP was excluded from the major IIPs and classified in the rare group of interstitial pneumonias in the revised 2013 ATS/ERS classification of IIPs [8]. Chronic immune reactions induced by bacterial or autoimmune stimuli play a major role in the development of EMZL. Although LIP rarely transforms into EMZL, four cases have been reported (Table 1) [9–12]. In the case reported here, the lesion in the left upper lobe, which was biopsied during bronchoscopy 9 years prior, was diagnosed as LIP. The diagnosis of EMZL was subsequently made on the basis of the specimen obtained after left upper lobe resection, supporting the diagnosis of malignant transformation. Since neither LIP nor EMZL has specific clinical symptoms or radiological findings, diagnosing these conditions can be difficult [13, 14]. Therefore, a diagnosis on the basis of pathology findings is necessary. The pathology findings for EMZL reveal that marginal zone cells originate in the pulmonary interstitium and grow along or infiltrate the interstitial lung tissue and bronchial submucosal epithelium, primarily destroying the pulmonary interstitium [15]. The bronchial wall remains intact, and as a result, residual bronchial shadows are often observed within the lesion [15]. Lymphoepithelial lesions (LELs), in which tumor cells infiltrate bronchial epithelium, are important diagnostic features of EMZL. LIP is characterized by dense interstitial lymphocytic infiltrates that expand and widen the interlobular and alveolar septa [6]. These infiltrates are generally polymorphous and consist of small lymphocytes mixed with varying numbers of plasma cells, immunoblasts, macrophages, and occasional histiocytes. EMZL cells typically express the B-cell-associated antigens CD20 + and CD79a + and are negative for CD3, CD5 and CD10 [10]. Interstitial lymphocytes associated with LIP are composed predominantly of T-cells and exhibit positive staining for CD3 [6]. Modalitiesfor diagnosing these conditionsinclude transbronchial biopsy, CT-guided needle aspiration biopsy, and surgical resection to obtain tissue samples for pathological diagnosis. However, the diagnostic rateachieves with less invasive sampling techniques, such as transbronchial biopsy and CT-guided needle aspiration biopsy, is low. Furthermore, owing to the presence of crush artifacts with these methods, a definitive diagnosis typically requires surgical resection [16]. Cryobiopsy has been reported to have high diagnosis rates because the samples are of higher quality, with no crush artifacts; this technique is thus expected to become a preferred diagnostic approach [17]. The appropriate treatment for EMZL is determined on the basis of the site of onset and the stage of the disease. Antibiotic therapy, currently the recommended first-line treatment, induces remission in most cases of gastric EMZL [18]. According to the current literature and the National Comprehensive Cancer Network guidelines, surgical resection is a therapeutic option for localized lesions [13]. However, recent findings have indicated that an observation-based approach without active treatment may also be a viable option for the management of patients with PPL [5, 19].It has been reported thatcompared withother treatment strategies,observation does not affect survivalorprognosis; this finding is believed to be due tothe indolent nature of PPL.The risk of relapse forextragastric EMZL has been reported to be high, and recurrence was also observed in the case reported here [14].There is no established observation period or additional treatment forextragastric MALT lymphoma, but careful follow-up should be conducted giventhe high relapse rate. In conclusion, we report the case of a patient who was diagnosed with EMZL more than 9 years after being diagnosed with LIP. Malignant transformation from LIP to EMZL has rarely been reported in the literature. In this case, a diagnosis could not be madevia bronchoscopy, so surgery was performed. Surgery is often selected for both diagnosis and treatment; however, another approach involves diagnosis via a less invasive biopsy technique, followed by a treatment plan that includes an observation-basedapproach.Even though progressive disease will eventually occur, necessitating some form of treatment, an initial observation-based strategy does not worsen the patients'clinical course and may be especially beneficial for asymptomatic patients, as they can avoid unnecessary treatment.

**Table 1:** Five patients were diagnosed with EZML, which transformed into LIP, as reported in the literature

Case	Country	Age	Sex	Clinical symptoms of LIP	Radiological findings of LIP	Background diseases	During the period from LIP to EMZL (years)	Clinical symptoms of EMZL	Radiological findings of EMZL	Diagnostic methods
①9)	South Korea	60	Female	Cough, dyspnea	Diffuse bilateral ground-glass opacity and consolidative lesions	None (iLIP)	6	Dyspnea	Extensive consolidative lesions and nodular lesions	CT-guided biopsy
②10)	USA	47	Female	None	Bilateral thin-walled cysts	Sjogren’s syndrome	4	None	New bilateral ground glass nodular opacities with air bronchograms	CT-guided biopsy
③11)	China	43	Female	Cough, sputum	Consolidation with air bronchogram	None (iLIP)	4.5	Chest pain	Extensive consolidative lesions with air bronchogram	Bronchoscopy
④12)	China	64	Female	Cough, dyspnea	Multiple cysts with multiple patchy and nodular opacities	Sjogren’s syndrome	None mention	Cough, fever	More patchy and nodular foci surrounded by ground-glass opacities	None mention
This case	Japan	45	Female	None	Consolidation with air bronchogram	None (iLIP)	9	None	Extensive consolidative lesions and nodular lesions	Surgery

*EMZL* Extranodal marginal zone lymphoma of MALT, *iLIP* idiopathic lymphocytic interstitial pneumonia

## Supplementary Information

Supplementary Material 1. The bronchoscopy findings revealed lymphocytic infiltration in the bronchial mucosa and macrophage exudation in the alveolar spaces.

## Data Availability

Not applicable.

## Abbreviations

MALT: Mucosa-associated lymphoid tissue
iLIP: Idiopathic lymphocytic interstitial pneumonia
EMZL: Extranodal marginal zone lymphoma
PPL: Primary pulmonary lymphoma
CT: Computed tomography
VATS: Video-assisted thoracoscopic surgery
PET: Positron emission tomography
CD: Cluster of differentiation
H. P: *Helicobacter pylori*
ATS/ERS: American Thoracic Society/European Respiratory Society
LEL: Lymphoepithelial lesion
