# Supplementary figures and images for: A case of pulmonary mucosa-associated lymphoid tissue (MALT) lymphoma in a patient with a history of idiopathic lymphocytic interstitial pneumonia (iLIP)
Source: Gen Thorac Cardiovasc Surg Cases. 2025 May 9;4:24. doi: 10.1186/s44215-025-00208-3 (PMC12065140; doi:10.1186/s44215-025-00208-3)

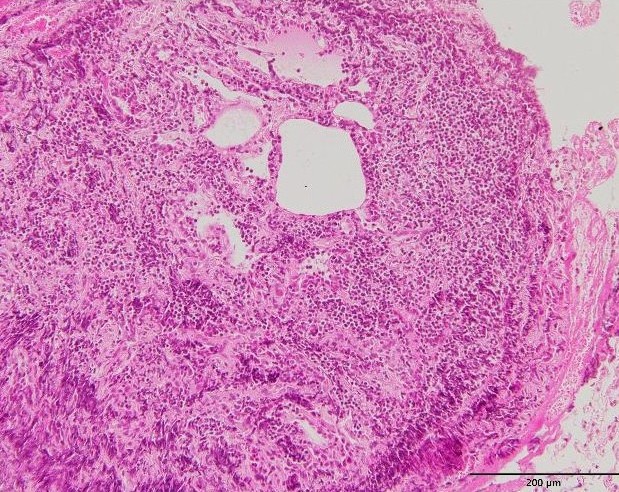

Supplement: Supplementary file 1 — Supplementary Material 1. The bronchoscopy findings revealed lymphocytic infiltration in the bronchial mucosa and macrophage exudation in the alveolar spaces. [file 44215_2025_208_MOESM1_ESM.jpg]
